# Mineral content variations between Australian tap and bottled water in the context of urolithiasis

**DOI:** 10.1002/bco2.168

**Published:** 2022-06-20

**Authors:** Michael Kwok, Stephen McGeorge, Matthew Roberts, Bhaskar Somani, Nicholas Rukin

**Affiliations:** ^1^ Department of Urology Redcliffe Hospital Redcliffe Queensland Australia; ^2^ Faculty of Medicine University of Queensland Brisbane Queensland Australia; ^3^ Department of Urology Royal Brisbane and Women's Hospital Brisbane Queensland Australia; ^4^ Faculty of Medicine University of Queensland Centre for Clinical Research Brisbane Queensland Australia; ^5^ University Hospitals Southampton NHS Trust Southampton UK

**Keywords:** bottled water, kidney stone disease, mineral composition, tap water, urolithiasis

## Abstract

**Objectives:**

The objective of this study is to investigate the variations in mineral content of tap drinking water across major Australian cities, compared with bottled still and sparkling water, and discuss the possible implications on kidney stone disease (KSD).

**Materials and Methods:**

The mineral composition of public tap water from 10 metropolitan and regional Australian cities was compared using the drinking water quality reports published from 2019 to 2021 by the respective water service utilities providers. Specifically, average levels of calcium, bicarbonate, magnesium, sodium, potassium, and sulphates were compared with published mineral content data from bottled still and sparkling drinking water in Australia.

**Results:**

The median or mean (depending on report output) mineral composition was highly variable for calcium (range 1.3 to 20.33 mg/L), magnesium (range 1.1 to 11.2 mg/L), bicarbonate (range 12 to 79 mg/L), sodium (range 3 to 47.1 mg/L), potassium (range 0.4 to 3.23 mg/L) and (sulphates range <1 to 37.4 mg/L). Calcium, magnesium and bicarbonate levels in tap water were lower than in bottled sparkling water. Consumption of 3 L/day of the most calcium rich tap water would fulfil 4.7% of the RDI, compared with 8.7% with bottled sparkling water. Consumption of 3 L of the most magnesium rich tap water would fulfil 8% of the RDI, compared with 13.6% with bottled sparkling water.

**Conclusion:**

The mineral content of tap drinking water varied substantially across major Australian city centres. Bottled sparkling water on average provided higher levels of calcium, bicarbonate and magnesium and may be preferred for prevention of calcium oxalate stones. These findings may assist counselling of patients with KSD depending on geographic location in the context of other modifiable risk factors and 24‐h urine analysis results.

## 1 INTRODUCTION

Kidney stone disease (KSD) is common and increasing in Australia,^1^ with an estimated annual incidence of 131 cases per 100 000.^2^ The prevalence of KSD ranges from 5% to 14% in the United States and Europe^3^, ^4^ and has doubled in the last 30 years. Stone recurrence is common with a risk of 53% at 5 years^3^ or a median recurrence of 15 episodes per 100 person‐years.^5^

Such a high tendency for recurrence has prompted prioritisation of preventative interventions and patient counselling. Increasing fluid intake is universally recommended to reduce recurrent KSD by 60% due to lower urinary supersaturation of calcium oxalate, calcium phosphate and uric acid.^6^ However, increasing fluid intake alone may not be sufficient, as the type of water consumed can affect urine composition and potentially influence stone formation. In patients with calcium stones, consumption of water with increased hardness, which contains higher levels of calcium and magnesium, is associated with increased urinary calcium and citrate excretion.^7^, ^8^

Tap water may be subject to regular and stringent testing and treatment, with addition of various substances to minimise microbiological growth and other potentially harmful contaminants, and its composition may be altered to reduce corrosion of distribution systems.^9^ Bottled mineral water are sourced from groundwater or springs and undergo limited treatment, whereas tap water can be derived from multiple sources, including artificial reservoirs, groundwater, lakes and rivers.^9^

Mineral composition of bottled drinkable water is known to vary substantially between countries.^3^ Despite accessibility of safe tap drinking water, an increasing worldwide trend towards consumption of bottled water has been observed.^10^ In the United States, bottled water consumption per capita has doubled to 138 L, and in France, this has increased from 6 L per person in 1940 to 141 L per person in 2015.^3^ Preference for bottled water is most commonly due to dissatisfaction with tap water taste and health risk concerns.^10^

While increasing water intake is universally recommended for stone prevention, it is not clear whether there are benefits to consumption of tap compared with bottled water and its implications on KSD. The objective of this study was to investigate the variations in mineral content of tap water across Australia and compare to the available published data on the average mineral content of bottled water. The study also aims to discuss the potential implications of mineral composition of drinking water on KSD.

## 2 MATERIALS AND METHODS

### 2.1 Tap water mineral content

We conducted a descriptive study investigating the tap water mineral content in 10 metropolitan and regional Australian centres (Brisbane, Gold Coast, Sydney, Canberra, Melbourne, Adelaide, Perth, Townsville, Cairns and Darwin). These centres were chosen due to the location of major urological centres and to ensure broad geographical coverage across Australia. The minerals of interest were calcium, magnesium, bicarbonate, sodium, potassium and sulphates. Data were obtained from drinking water quality reports published by the respective water utilities service providers from 2019 to 2021, and they were also contacted where further data were required (see supporting information Table S1).^11^, ^12^, ^13^, ^14^, ^15^, ^16^, ^17^, ^18^, ^19^, ^20^

### 2.2 Bottled water mineral content

We compared our findings to the average mineral content of bottled still and bottled sparkling mineral waters available in the two main supermarket chains in Australia. These data were extracted from a previously published multicontinental descriptive study conducted in 21 countries, which was also the first study to investigate the mineral composition of bottled drinkable water in the context of KSD.^3^

### 2.3 Outcome measures

The primary outcome was the mineral composition of tap water and bottled water.

### 2.4 Statistical analysis

A descriptive comparative analysis was performed on the basis of mean/median mineral content values extracted from data sources. Formal comparative analysis of tap and bottled water data was not possible.

## 3 RESULTS

There is a vast difference in water mineral composition of Australian tap water, based on location alone. The median/mean mineral composition of calcium ranged from 1.3 to 20.33 mg/L, magnesium ranged from 1.1 to 11.2 mg/L, bicarbonate ranged from 12 to 79 mg/L, sodium ranged from 3 to 47.1 mg/L, potassium ranged from 0.4 to 3.23 mg/L and sulphates ranged from <1 to 37.4 mg/L. These results are detailed in Table 1.

**TABLE 1:** Mineral composition of tap water across Australia, compared with average mineral content in bottled still and sparkling water available in Australia

	Median/mean mineral composition (mg/L)					
	Calcium	Magnesium	Bicarbonate	Sodium	Potassium	Sulphates
Brisbane	20.33	11.2	79	43	2.9	26
Gold coast	16	2.7	56	17		
Sydney^a^	14.1	4.3	40.3	15	2.3	8.1
Canberra	14.3	1.43	53	3	0.7	4
Melbourne	3.6	1.1	15.9	4	0.58	<1.5
Adelaide	19.7	10	55	47.1	3.23	37.4
Townsville	10	2		14	2	<1
Cairns	1.3	1.05	12	4.8	0.7	0.7
Darwin	5	5	36.6	3	0.4	<0.3
Perth			61.4	33.3		
Bottled still water^b^	18	4	130	6.6	0.7	6.6
Bottled sparkling water^b^	37.8	19	233	7	1	16
Recommended dietary intake (mg/day)	1000–1300	255–420	n/a	460–920	2800–3800	n/a

^a^ Median values for Sydney extrapolated from the 10‐90th percentile range.

^b^ Stoots et al.^33^

**FIGURE 1:**
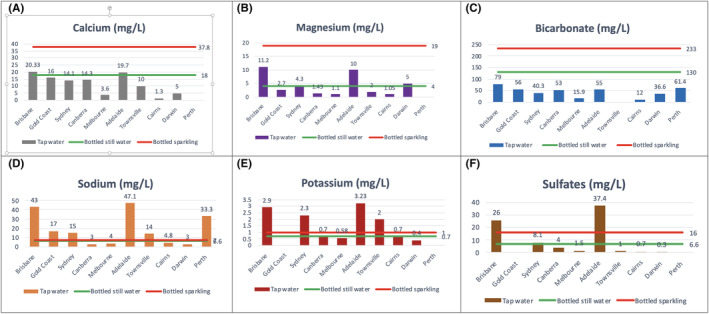
(A–F) Mineral composition of tap water across Australia, compared with average mineral content in bottled still and sparkling water available in Australia (Stoots et al.^33^)

In bottled sparkling water available in Australia, the average calcium, magnesium and bicarbonate levels were 37.8, 19, and 233 mg/L, respectively, which is higher compared with bottled still water and tap water. Comparisons of the mineral content of tap and bottled water are detailed in Figure 1.

The Australian and New Zealand Governments and the National Health and Medical Research Council (NHMRC) have published Recommended Dietary Intake (RDI) values for calcium and magnesium and Adequate Intake (AI) values for sodium and potassium.^21^ These are listed in Table 1:. The calcium RDI for adults is 1000–1300 mg,^21^ and therefore, consumption of 3 L/day of the most calcium rich tap water would fulfil 4.7% of the RDI, compared with 8.7% with bottled sparkling water. For magnesium the RDI is 255–420 mg/day for most adults^21^ and therefore consumption of 3 L of the most magnesium rich tap water would fulfil 8% of the RDI, compared with 13.6% with bottled sparkling water. The adequate intake (AI) values for sodium are 460–920 mg/day for adults,^21^ and therefore, consumption of 3 L of the most sodium rich tap water would fulfil 15.6% of the AI, compared with 2.3% with bottled water. The AI for potassium is 2800–3800 mg/day for adults,^21^ and the contribution from either tap or bottled water is minimal.

## 4 DISCUSSION

Previous studies noted substantial variation in the mineral content of bottled water in Europe^22^ and also globally across 21 countries.^3^ Globally, the median calcium and bicarbonate levels of sparkling water varied by factors of 7.4 and 57.3, respectively, and for still water 18.7 and 12.6, respectively.^3^ For instance, some bottled waters in Switzerland contain up to 579 mg/L of calcium (Abdelbodner Cristal), which is a significant contribution to the overall daily intake.^3^ These studies focused on the potential impacts of water mineral content on kidney stone formation and also bone health and cardiovascular risk and consequently recommended counselling patients on an individual level regarding water intake.^3^, ^22^ We investigated the mineral content of tap water in Australia. Although the safety standards of tap drinking water in Australia are regulated by state legislation and nationally published guidelines,^23^ in our study, we nevertheless noted variations in the mineral content.

Calcium levels in tap water varied by a factor of 15.6, where levels were highest in Brisbane with 20.33 mg/L and the lowest in Cairns with 1.3 mg/L. Bottled still and sparkling water had average calcium levels of 18 and 37.8 mg/L, respectively. Approximately 85% of kidney stones are predominantly calcium oxalate and/or calcium phosphate in composition and most commonly occur in the setting of idiopathic hypercalciuria.^24^ Hypercalciuria exceeding 200 mg per day increases stone formation risk.^24^ However, high dietary calcium intake decreases symptomatic stone formation, which is seemingly counterintuitive. A prospective cohort study involving 45 619 participants reported a 34% reduction in stone risk in the group with the highest mean daily dietary calcium intake of 1326 mg.^25^ The reduced KSD risk with increased dietary calcium may be explained by increased binding of calcium to oxalate in the gut, reducing oxalate absorption and consequently reducing hyperoxaluria.^26^ Nevertheless, given the relatively low levels of calcium in Australian tap water, it likely has limited influence on overall dietary intake. Perhaps those who have inadequate calcium intake from other dietary sources may want to consider bottled water as opposed to tap water to increase dietary intake. Given the mechanism by which dietary intake may assist in reducing KSD, the timing of calcium rich water consumption with meals may potentially be beneficial and an area worth further investigating. We also noted that the EAU and AUA recommendations for calcium intake in stone formers were 1000–1200 mg, which is consistent with the general Australian RDI of 1000–1300 mg.^27^, ^28^

Bicarbonate levels in tap water varied by a factor of 5.12, where levels were highest in Perth with 61.4 mg/L and lowest in Cairns with 12 mg/L. Bottled water tended to be a more abundant source, and even comparing to the most bicarbonate rich tap water, bottled still and sparkling water had a 2.1‐ and 3.8‐fold higher bicarbonate content, with 130 and 233 mg/L, respectively. Bicarbonate provides an alkaline load, thereby increasing urinary pH and urinary citrate excretion, preventing aggregations of calcium oxalate.^3^, ^29^ However, in calcium phosphate stones where treatment aims to lower urinary pH, increasing dietary bicarbonate would be counterproductive.^3^

Magnesium levels in tap water varied by a factor of 10.7, with the highest levels found in Brisbane with 11.2 mg/L and the lowest levels in Cairns with 1.05 mg/L. Of note is that the majority of the US population have insufficient intake.^30^ While there is no overall consensus as to whether magnesium supplementation reduces stone formation,^3^ it does increase urinary citrate excretion in those with magnesium deficiency.^8^ Furthermore, in a similar mechanism of calcium binding to oxalate in the gut, increasing oral magnesium intake may similarly decrease oxalate absorption and consequently reduce hyperoxaluria.^8^ Epidemiological studies have also observed an inverse relation with ischemic heart disease, cardiac arrythmias and sudden death.^30^

In contrast with calcium, bicarbonate and magnesium, the levels of sodium in tap water tended to be higher than bottled water and varied by a factor of 15.7, with the highest levels in Adelaide with 47.1 mg/L and the lowest in Canberra with 3 mg/L. Bottled still and sparkling water provided 6.6 and 7 mg/L, respectively. In the typical North America diet, sodium intake in the form of salt, usually exceeds the RDI and is estimated to range from 4000 to 6000 mg/day.^30^ In stone formers, increasing dietary sodium increases hypercalciuria and hypocitraturia, both unfavourable for recurrent KSD.^31^ Excessive sodium intake also contributes to hypertension and adverse cardiovascular disease risk.^30^ We noted consistency in the EAU and AUA recommendations for limiting sodium intake in stone formers and the general Australian adequate intake values, but the upper limits varied (4000–5000, 2300 and 460–920 mg, respectively).^27^, ^28^

While both tap and bottled water in Australia contribute minimally to overall potassium intake, increasing intake from other sources such as fruits and vegetables can reduce kidney stone risk by 35%–56%.^32^

In terms of limitations, this is a descriptive study by design, and consequently, we were unable to draw conclusions about causation. Given the geographical locations included in our study, the results may not be generalisable outside Australia. There was a small amount of missing data despite contacting the water service providers, and some locations only reported mean values for the mineral concentrations. However, where both mean and median were reported, values were very similar. For Sydney, median values were inferred from the reported range. While comparisons were made with bottled water, these are average values across brands available in Australia as per manufacterurer reports and not verified with an independent laboratory. Patients should be encouraged to note the mineral content of the individual brands being consumed. Further research with water analysis at a single laboratory would yield more reliable results and enable investigation of any other minerals suspected to contribute to KSD. Furthermore, future studies could correlate mineral content findings with geographical differences in KSD incidence.

In summary, depending on geographical location, the preferred water varies; for example, calcium oxalate stone formers in Brisbane may be better with tap water compared with a similar index patient in Melbourne. One could hypothesise that in patients with calcium oxalate stones, an ideal water would be high in calcium, magnesium and bicarbonate, and based on our data, bottled sparkling water makes a good long‐term option. There are multiple competing/environmental factors which affect stone formation which may warrant future investigation. Bottled sparkling water on average in Australia, compared with tap water, provides a more abundant and clinically significant source of calcium, bicarbonate and magnesium. Counselling patients at risk of recurrent KSD should include discussion not just about the volume of fluid but also the type of water. The influence of water intake and KSD needs to be interpreted in the context of patient factors, such as the type of recurrent stone former and 24‐h urine analysis, other dietary intake and nondietary risk factors. Furthermore, clinicians will also need to consider the other health benefits and harms of minerals found in water, not just in relation to KSD.

## CONFLICT OF INTEREST

The authors declare that they have no conflicts of interest.

## AUTHOR CONTRIBUTIONS

Concept and design: NR, BS. Data collection and analysis: MK, NR, MR. Drafting of the manuscript: MK, NR, MR, SM. All authors reviewed the results and approved the final version of the manuscript.

## Supporting information


**Table S1.** Water utilities service providers
