# Supplementary material for: Mineral content variations between Australian tap and bottled water in the context of urolithiasis
Source: BJUI Compass. 2022 Jun 20;3(5):377–82. doi: 10.1002/bco2.168 (PMC9349584; doi:10.1002/bco2.168)
Supplement: Supplementary file 1 — Table S1. Water utilities service providers [file BCO2-3-377-s001.docx]

**Variations in mineral content in Australian tap and bottled water: Possible impact on urolithiasis?**

**Supplementary table 1:** Water utilities service providers

| **Location** | **Provider** | **Website** |
| --- | --- | --- |
| Brisbane | UrbanUtilities | https://urbanutilities.com.au/business/business-services/water-quality-data |
| Gold Coast | Gold Coast Water and Waste | https://www.goldcoast.qld.gov.au/Services/Water-sewerage/Water-quality/Drinking-water-quality |
| Sydney | WaterNSW | https://www.waternsw.com.au/water-quality/quality/reports |
| Canberra | Icon Water | https://www.iconwater.com.au/My-Home/Water-quality.aspx |
| Melbourne | Melbourne Water | https://www.melbournewater.com.au/about/strategies-and-reports/water-quality-annual-report |
| Adelaide | SA Water | https://www.sawater.com.au/water-and-the-environment/safe-and-clean-drinking-water/your-tap-waters-quality-and-testing/your-drinking-water-profile |
| Perth | Water Corporation | https://www.watercorporation.com.au/About-us/Our-performance/Drinking-water-quality |
| Townsville | Port of Townsville | https://www.townsville-port.com.au/environment/monitoring/drinking-water-monitoring-program/ |
| Cairns | Cairns Regional Council | https://www.cairns.qld.gov.au/water-waste-roads/water-supply-and-use/sources-and-supply |
| Darwin | PowerWater | https://www.powerwater.com.au/about/what-we-do/water-supply/drinking-water-quality/past-drinking-water-quality-reports |
